# Making sense of human interaction benefits from communicative cues

**DOI:** 10.1038/s41598-020-75283-3

**Published:** 2020-10-22

**Authors:** Dimitrios Kourtis, Pierre Jacob, Natalie Sebanz, Dan Sperber, Günther Knoblich

**Affiliations:** 1 Psychology, Faculty of Natural Sciences, University of Stirling, Stirling, FK9 4LA Scotland UK; 2 Department of Cognitive Science, Central European University, Budapest, Hungary; 3 Institut Jean Nicod (CNRS, EHESS, ENS), Paris, France

**Keywords:** Cognitive neuroscience, Social neuroscience, Psychology

## Abstract

We investigated whether communicative cues help observers to make sense of human interaction. We recorded EEG from an observer monitoring two individuals who were occasionally communicating with each other via either mutual eye contact and/or pointing gestures, and then jointly attending to the same object or attending to different objects that were placed on a table in front of them. The analyses were focussed on the processing of the interaction outcome (i.e. presence or absence of joint attention) and showed that its interpretation is a two-stage process, as reflected in the N300 and the N400 potentials. The N300 amplitude was reduced when the two individuals shared their focus of attention, which indicates the operation of a cognitive process that involves the relatively fast identification and evaluation of actor–object relationships. On the other hand, the N400 was insensitive to the sharing or distribution of the two individuals’ attentional focus. Interestingly, the N400 was reduced when the interaction outcome was preceded either by mutual eye contact or by a perceived pointing gesture. This shows that observation of communication “opens up” the mind to a wider range of action possibilities and thereby helps to interpret unusual outcomes of social interactions.

## Introduction

Making sense of the social world involves not only interacting appropriately with others but also understanding interactions among others as a third-party observer. Such understanding may be facilitated by the more or less conventional role that verbal and non-verbal signals play in these interactions. Among these signals, mutual eye gaze and pointing have a particular importance. Both emerge early in human infancy before language and keep playing a major role in adult verbal and non-verbal communication.

Two- to five-day-old newborns have a preference for an agent’s face making eye contact with them rather than displaying an averted gaze^1^. Ten-month-olds understand the significance of mutual eye contact between two individuals and expect a person to look at her partner during conversation^2^. After seeing an agent make direct eye contact with them, 8-month-olds interpret the agent’s gaze shift as a referential action^3^. Twelve-month-old human infants understand the communicative import of imperative pointing and point helpfully for another’s benefit^4^. Fourteen-month-olds keep track of common ground when faced with an agent’s act of pointing^5^. Fifteen-month-olds point to request information from knowledgeable adults^6^. Finally, eighteen-month-olds understand that joint attention is necessary for two agents to share a common goal and engage in joint action^7,8^.

In adult interactions, all verbal or non-verbal communicative signals tend to have an open-ended range of contextually determined interpretations^9^. Mutual eye contact between two individuals may often provide evidence that their interaction is communicative, but it leaves the content of the communicated information wide open. In and of its own, it can convey diverse contextual meanings^10^. It can also be used to coordinate turn-taking during conversation^11^ and can modulate the tendency to follow others' gaze^12^. In the context of joint action between two agents, pointing is typically taken by adults (and children) to result in joint attention. Whether used on its own or integrated with speech, it can, however, convey a much wider range of interpretations^13–16^.

Here, we investigated how observing communicative signals between two agents determines expectations of interaction outcomes. We examined a situation in which participants viewed a sequence of three frames depicting two interacting individuals seated at a table with two objects. First, one actor (the ‘communicator’) looked the other actor (the ‘recipient’) in the eyes and the latter either looked back or kept his/her eyes closed, with equal probability. Second, the communicator shifted his/her gaze and pointed at one of two objects on a table, and the recipient either saw the pointing gesture or had his/her eyes closed, with equal probability. Finally, the two actors either looked at the same or at different objects, with equal probability. We hypothesized that, as the action sequences evolved, the different communicative interactions observed would modulate perception and interpretation of the observed action sequence.

To address this hypothesis, we recorded the electroencephalograms (EEG) of the participants, in order to investigate their brain activity with high temporal precision. Our analyses targeted two event related potentials (ERPs) of negative polarity that we expected to record during the observation of the final scene. The first ERP of interest, termed N300, peaks between around 250 and 350 ms after the onset of a visual image; it is considered to be specific to the processing of pictorial, non-verbal stimuli^17,18^ and to reflect relatively fast processes related to the difficulty of identifying an object within the context of a visual scene^19^. The second ERP of interest is the extensively studied N400, which peaks between 350 and 550 ms after the onset of stimulus (e.g. word, music note, action etc.) and is considered to reflect the operation of a multimodal comprehension system, which enables the construction of meaning through the binding of current contextual information and previous experience^20,21^. The N400 is present in conditions of reduced awareness^22^ and it is enlarged after the detection of semantic irregularities, including irregularities in action sequences^23^.

We hypothesized that the implicit interpretation of the outcome of the action sequences in our experiment would take place in two stages, the first of which would involve the identification and categorization of the content of the final visual scene in relation to image-based representations in long-term memory (indexed by the N300 amplitude^24,25^). We expected that the N300 amplitude would depend on whether the two actors attended to the same or to different objects and that it might be smaller in the former condition, because it is more common for two people who are seated around a table to share the focus of attention.

We also hypothesized that the second stage of interpretation of the outcome of the action sequences would involve the binding of the content of the final visual scene with information that had been collected earlier during the observation of the first two visual scenes in the action sequence^20^. We expected that the difficulty of making sense of a given interaction outcome would be reflected in the N400 amplitude, which should be enlarged when the absence of communicative signals makes the interpretation of the outcome a more complex process^23,25^. With regard to effects of observing joint attention, we considered two contrasting hypotheses. If earlier observation of communicative signals generates implicit expectations of joint attention between the observed agents, then deriving the meaning of a visual scene in which the two actors attend to two different objects should be a more complex process compare to deriving the meaning of a visual scene in which the two actors attend the same object; consequently, the N400 amplitude should be larger in the latter case. If, on the other hand, the expectations generated by the observation of communicative signals take into account the relatively open range of interpretations and behaviours that may ensue after mutual gaze and pointing in adult communication, then, regardless of whether the actors end up attending to the same or to different objects, their behaviour should not be seen as semantically anomalous and the N400 amplitude should be similar in both conditions.

## Methods

### Participants

EEG data were recorded from twenty-four healthy volunteers (21 right-handed, 12 females, age = 27.1 ± 4.8 years). All participants had normal or corrected-to-normal vision and provided their informed consent after full explanation of the study. All methods were carried out in accordance with relevant guidelines and regulations. The study was approved by the United Ethical Review Committee for Research in Psychology (EPKEB) in Hungary.

### Experimental setup and procedure

The experiment was run in a quiet, normally illuminated room. The participants were seated comfortably in front of a table with their index fingers placed on pre-designated keys on a standard keyboard. Stimuli were presented centrally on a 17’’ PC screen, located approximately 80 cm from the participants.

**Figure 1:**
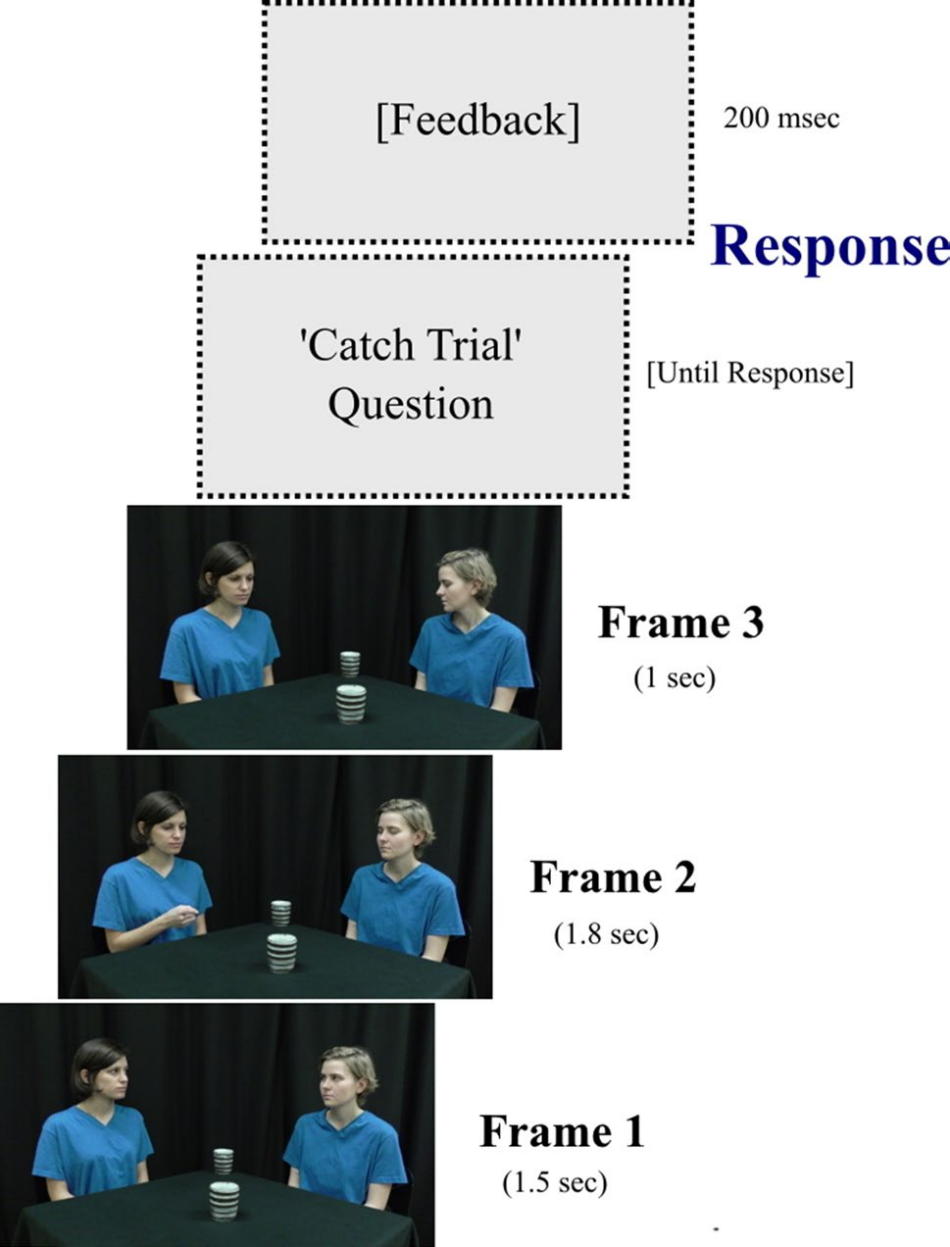
Example of an experimental trial. In frame 1, the recipient has her eyes open (i.e. mutual eye contact). In frame 2, the recipient has her eyes closed (i.e. no perception of the pointing gesture). In frame 3, the recipient does not look at the object to which the communicator had previously pointed. In 16% of the trials, the frame sequence was followed by a question that referred to one of the preceding frames. Feedback ([Correct] or [Incorrect]) was given after each response.

Each trial consisted of a sequence of three frames (27 × 15 cm) (Fig. 1). The first frame was displayed for 1.5 s, the second frame for 1.8 s and the third frame for 1 s. The durations of displays were selected with the aim of approximating a natural sequence of events and providing enough time to the participants to explore the relevant aspects of each scene.

The first frame depicted two actors (either a couple of females or a couple of males) seated at adjacent sides of a rectangular table that was covered by a black tablecloth (Fig. 1). The actors were confederates of the experimenter and they provided their informed consent for the use of the photos as experimental stimuli and for publication in an on-line open access academic journal, after full explanation of the purpose of the study. The photos were taken and processed by the first author of the present article at the CEU Social Body and Mind (SOMBY) lab. Two nearly identical Japanese clay mugs were placed along one diagonal of the table at equal distances from each person. The two actors had their heads turned towards the midline, in order to face each other. The eyes of one actor (the “communicator) were always open, whereas the eyes of the other actor (the “recipient”) were either open or closed with equal probability. Thus, the two actors established mutual eye contact in 50% of the trials. The second frame depicted the communicator looking and pointing at one of the objects using his/her right index finger. The eyes of the recipient were either open or closed with equal probability. Thus, the recipient perceived the pointing gesture in 50% of the trials. The third frame depicted the communicator looking at the previously pointed object, whereas the recipient looked either at the same or at the other object with equal probability. Thus, the two actors looked at the same object in 50% of the trials. This resulted in eight experimental conditions (Table 1).

**Table 1:** Experimental conditions.

Condition	Mutual eye contact in frame 1	Recipient perceives the pointing gesture in frame 2	The two actors look at the same object in frame 3
1	Yes	Yes	Yes
2	Yes	Yes	No
3	Yes	No	Yes
4	Yes	No	No
5	No	Yes	Yes
6	No	Yes	No
7	No	No	Yes
8	No	No	No

The participants’ task was to attend to the frames. In order to acquire an index of their level of attention, “catch trials” were randomly interspersed within the “standard trials”. The catch trials differed from the standard trials in that the frame sequence was followed by a question, which referred to one of the preceding frames with equal probability. Specifically, the questions were:

- Did the recipient look the communicator in the eyes? (Reference to frame 1).
- Did the recipient perceive the pointing gesture? (Reference to frame 2).
- Did both persons look at the same object? (Reference to frame 3).

The participants were asked to answer each question with “yes” or “no”, by pressing one of the designated keys (“m” and “z”). The correspondence between the two keys and the answers were counterbalanced across participants. The question remained on the screen until the participants responded. After each response, the participants received feedback, which consisted of the words ‘Correct’ or ‘Incorrect’ and was visible for 200 ms. Thus, the emphasis was put on the accuracy of the response. The participants were explicitly instructed to take as much time as necessary in order to provide an accurate response.

The experiment consisted of eight blocks. Each block had an approximate duration of 7 ½ minutes and consisted of 72 trials, 12 of which were catch trials. The inter-trial interval was randomized between 2.5 and 3.5 s. In half of the blocks, the communicator was on the left side of the screen and in the other half on the right side. The blocks were presented in an alternating fashion (communicator on the left, then communicator on the right and so on) and their order was counterbalanced across participants. The experimental blocks were preceded by two practice blocks of approximately 4 min each, one with the communicator on the left side and another one with the communicator on the right side.

### Data acquisition

Participants' key presses were recorded using a standard computer keyboard. EEG was recorded continuously from the participants using carefully positioned nylon caps (Acticap, BrainProducts GmbH, Germany) with 63 electrodes, arranged according to an extended version of the 10–20 system. All electrodes were referenced to the right mastoid during recording. Vertical and horizontal eye movements were monitored by pairs of electrodes, positioned above and below the middle of the left eye and lateral to each eye, respectively. Electrode impedance was kept below 20 kΩ. EEG and EOG signals were amplified with a band-pass filter of 0–250 Hz by two BrainAmp DC Amplifiers (Brain Products GmbH, Gilching, Germany) and sampled at 500 Hz.

### Data processing and analysis

EEG data processing was performed using Brain Vision Analyzer 2.1.0 (Brain Products GmbH, Germany). EEG data were first re-referenced to the mean of both mastoid electrodes. The data were filtered using a low cut-off filter of 0.1 Hz (24 dB/octave) and a high cut-off filter of 40 Hz (24 dB/octave) to remove the influence of slow drifts and excessive high-frequency noise, respectively. In addition, a notch filter at 50 Hz was used to suppress power line interference. Ocular Correction was performed on unsegmented data using the Gratton & Coles algorithm^26^, as implemented in BrainVision Analyzer 2. Then, the data were segmented offline into epochs from 300 ms before until 1200 ms after the onset of frame 3. Semi-automatic artefact rejection was performed before averaging in order to remove individual trials containing remaining vertical eye movements or other EEG-related artefacts. An epoch was rejected when the difference between the maximum and minimum value at a single channel exceeded 100 µV. Averages were constructed separately for each condition and each participant. Finally, the data were baseline corrected relative to the time period from 200 ms before until the onset of frame 3.

The amplitudes of the ERPs of interest were evaluated on a peak-to-peak basis. The N300 was quantified as the mean amplitude between 285 and 315 ms after frame onset with respect to the mean amplitude between 215 and 245 ms after frame onset, whereas the N400 was quantified as the mean amplitude between 370 and 400 ms after frame onset with respect to the mean amplitude between 490 and 520 ms after frame onset. The selection of the time intervals of analyses were based on the aggregate grand average from trials (AGAT)^27^. Typically, the N300 as well as the N400 exhibit a topographical distribution along the midline of the scalp, although the distribution along the anterior–posterior axis varies across studies. Thus, we selected three regions of interest (ROIs) along the midline: an anterio-frontal ROI (mean activity from electrodes AFz, AF3, AF4, Fz, F1 and F2), a fronto-central ROI (mean activity from electrodes FCz, FC1, FC2, Cz, C1 andC2) and a centro-parietal ROI (mean activity from electrodes CPz, CP1, CP2, Pz, P1 and P2).

In order to get a complete picture of all the relevant cognitive processes, we also analysed the amplitude of the P300 potential, which is considered an indirect index of working memory processes^28–30^. The P300 was quantified as the mean amplitude between 370 and 400 ms after frame onset with respect to the mean amplitude between 285 and 315 ms after frame onset in the same ROIs as the N300 and N400 potentials.

Statistical analyses were performed by means of repeated measures 3 × 2 × 2 × 2 ANOVAs (Greenhouse–Geisser corrected when assumption of sphericity was violated) with factors: ROI (anterio-frontal vs. fronto-central vs. centro-parietal), Mutual Eye Contact in frame 1 (‘yes’ vs. ‘no’), Perception of Pointing in frame 2 (‘yes’ vs. ‘no’) and Joint Attention in frame 3 (‘attending to the same object’ vs. ‘attending to different objects’). Post-hoc multiple comparisons (Bonferroni correction applied) were performed by means of paired samples t-tests.

## Results

### Behavioural analysis

We considered the number of erroneous responses in the catch trials as a tentative index of the level of attention and task engagement. The mean number of errors were 9.46 ± 5.34 (range 1–19 in 96 catch trials). More specifically, there were on average 4.46 ± 2.71 errors to the question referring to frame 1 (range 1–10), 3.75 ± 2.83 errors to the question referring to frame 2 (range 0–9) and 1.25 ± 1.51 errors to the question referring to frame 3 (range 0–5). This corresponds to an overall response accuracy of 90.1 ± 9.9% (range 80.21–98.96%), which shows that our participants were quite accurate overall in recollecting, and therefore attending to, crucial features of the interaction.

### EEG analysis

**Figure 2:**
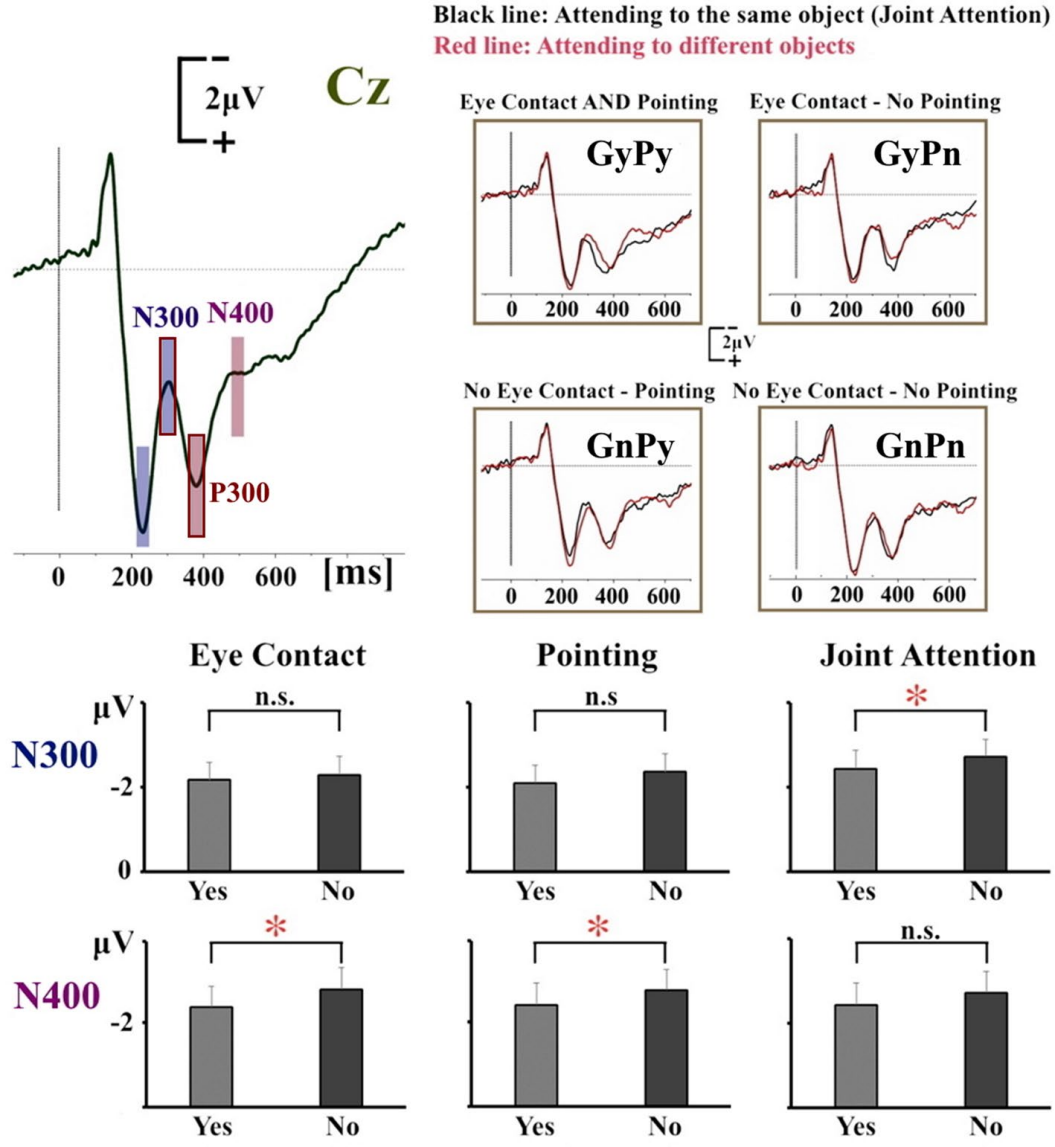
ERP waveforms and mean amplitudes. Top Left Panel: Grand Average ERP waveforms of all conditions from representative electrode Cz (apex of the head). The blue and violet rectangles correspond to the selected time intervals for the peak-to-peak amplitude analysis of the N300 and N400, respectively. The rectangles with the red outlines correspond to the selected time intervals for the peak-to-peak amplitude analysis of the P300. Top Right Panel: Grand Average ERP waveforms separately for different conditions. The vertical lines indicate the onset of frame 3. Lower Panel: N300 and N400 amplitudes—the asterisks indicate statistically significant differences.

The statistical evaluation of the N300 amplitude (Fig. 2) revealed a significant main effect of ROI (F(2,46) = 17.0, p < 0.001, *n*_*p*_^*2*^ = 0.425), because the N300 amplitude was larger over fronto-central areas. Importantly, there was a significant main effect of Joint Attention (F(1,23) = 5.6, p = 0.027, *n*_*p*_^*2*^ = 0.195), because the N300 was larger when the participants observed the two actors attending to different objects. There was also a significant 3-way ROI × Mutual eye contact × Perception of pointing interaction (F(2,46) = 7.0, p = 0.006, *n*_*p*_^*2*^ = 0.234), but it is not possible to provide a reliable interpretation because none of the related 2-way interactions were statistically significant. No other main effect or interaction reached statistical significance (ps > 0.14).

The statistical evaluation of the N400 amplitude (Fig. 2) revealed a significant main effect of ROI (F(2,46) = 26.4, p < 0.001, *n*_*p*_^*2*^ = 0.534), because the N400 amplitude was larger primarily over anterio-frontal, but also over fronto-central areas, and much smaller over centro-parietal areas. The main effect of Joint Attention was not significant (p = 0.201), but there was as a significant ROI x Joint Attention interaction (F(2,46) = 6.5, p = 0.011, *n*_*p*_^*2*^ = 0.220). Post-hoc paired t-tests showed that there was no difference over anterio-frontal areas (p = 0.708), over fronto-central areas (p = 0.387) or over centro-parietal areas (p = 0.033) (given the adjusted level of significance of 0.017 to account for multiple comparisons). More importantly with regards to the purpose of this study, there was a significant main effect of Mutual Eye Contact (F(1,23) = 7.5, p = 0.012, *n*_*p*_^*2*^ = 0.246), because the N400 was larger when the two actors had not engaged in mutual eye contact in frame 1. Additionally, there was a significant main effect of Perception of Pointing (F(1,23) = 5.8, p = 0.025, *n*_*p*_^*2*^ = 0.201), because the N400 amplitude was larger when the recipient had not perceived the pointing gesture in frame 2. No other interactions were statistically significant (ps > 0.065).

The statistical evaluation of the P300 amplitude (Fig. 2) revealed a significant main effect of ROI (F(2,46) = 13.6, p = 0.001, *n*_*p*_^*2*^ = 0.372), because the P300 amplitude was larger over centro-parietal areas. No other main effects or interactions were statistically significant (ps > 0.069).

## Discussion

We investigated whether the observation of non-verbal communicative signals facilitates the interpretation of uncommon interaction outcomes that involve two individuals jointly attending to the same object or to different objects. The EEG analysis identified two ERPs, namely the N300 and the N400, that were elicited during the observation of the final visual scene and are typically related to the on-line semantic processing of individual stimuli or sequences of pictorial, non-verbal stimuli^17,25^. These are sometimes collectively referred to as the N300/N400 complex^31^. Interestingly in our study, the amplitude of the N300 was smaller when the two actors attended to the same object. However, it was insensitive to the observation of the preceding communicative signals, despite the fact that the attention of the observer was guided towards the observation of these signals via the inclusion of the “catch trials”. The N300 is believed to reflect the matching of visual input to long-term memory representations^24^. It is enlarged when an object is perceived as incongruous within the context of a visual scene^19,32,33^. The incongruity in the present study did not arise from object-scene relationships, but from interpersonal attentional relationships that may be expected to a different degree, when two individuals are seated close to each other and occasionally interacting with each other. The enlarged N300 that occurred when the two actors attended different objects points to the operation of a relatively fast categorization process that considers the sharing of attentional focus between the two actors as the most common or perhaps the most congruous event^34^.

In contrast to the N300, the N400 during the processing of the final visual scene was unaffected by whether or not the two actors shared attention, but it was smaller when the two actors had previously communicated via mutual eye contact or via a pointing gesture. The N400 is typically enlarged in relation to semantic incongruities in verbal and non-verbal communication, and also in mathematics, action sequences and interpersonal coordination^25,32,35–37^. It is believed to reflect the operation of a multimodal comprehension system that binds current contextual input with previous experiences in order to derive the meaning of any given stimulus^20,21,38^. Hence, the N400 in the present study may reflect the difficulty in extracting meaning from the final visual scene in relation to expectations that were generated by the observation of the preceding events. This interpretation is supported by the presence of the P300 potential, which preceded the N400 and it is considered an indirect index of accessing working memory processes^28–30^. The similar N400 amplitude when the two actors attended the same object or different objects supports the hypothesis that the absence of joint attention was not considered as a semantically anomalous event within the context of the present interaction, but rather as a possible outcome of an interaction between two agents. Our findings suggest that observing any communicative interaction between two agents triggers implicit anticipations of a wide range of possible interaction outcomes, which allows a person to make sense of typically unexpected situations.

Making sense of a social situation from the point of view of a third-party observer could be cognitively demanding, because the observer does not have access to the internal states of others and often has to rely only on contingencies and communicative signals that are not directed towards the observer^39^. The ability to interpret communicative signals, such as mutual eye contact and pointing gestures, and to predict the behaviour of others, develops early in human life^2,4^. In many cases, communicative signals provide cues of the establishment of a psychological common ground between two agents and of their subsequent cooperative behaviour and/or joint attentional interactions^8,12^.

The intriguing and seemingly paradoxical finding reported in the present study is that there seems to be a general expectation for joint attention that does not depend on prior observation of communication between two individuals (N300). However, after the communicative signals have been taken into account (N400), expectations of joint attention turn out to be no stronger than expectations of divided attention. Mutual eye contact is one of the most common referential communicative signals, but its content is unknown to an observer and can only be inferred on the basis of preceding or succeeding events. For example, one may perceive mutual eye contact between two basketball players as a signal of communication, but in order to infer its content, one would need to observe what follows next (e.g. one player passing the ball to the other) and/or have knowledge of what preceded the mutual eye contact (e.g. a mishandling of the ball). Similarly, an observed pointing gesture may have a referential or an imperative character, but it is also subject to a wide range of interpretations. Our findings suggest that mutual eye contact between two individuals as well as perceived pointing gestures are indeed interpreted as instances of communication in the eyes of a passive observer. However, instead of narrowing down the space of possible interpretations, they expand the space of interaction outcomes that is considered to be interpretable. Further research is required to determine the types of communicative interactions to which the observed expansion of expectations applies to. For example, future experiments could investigate situations in which the recipient would behave in an ostensibly uncooperative or unreliable manner. It is conceivable that the observation of those type of interactions would not generate expectations of joint attention even if it is preceded by mutual eye contact.

To conclude, the analysis of the observer’s electroencephalogram showed that the processing of interaction outcomes takes place in two stages. First, the observer categorizes and possibly evaluates present actor–object relationships based on representations stored in long-term memory. Then, the observer accesses information in working memory in order to bind the present scene with the events that took place earlier in the interaction and makes sense of all possible interaction outcomes. Importantly, our findings suggest that observation of communicative signals, such as mutual eye contact and pointing gestures, does not automatically generate expectations of joint attention but rather opens up the mind to a range of possible interaction outcomes.
